# Efficacy of CytoSorb in a Pediatric Case of Severe Multisystem Infammatory Syndrome (MIS-C): A Clinical Case Report

**DOI:** 10.3389/fped.2021.676298

**Published:** 2021-06-11

**Authors:** Gabriella Bottari, Valerio Confalone, Nicola Cotugno, Isabella Guzzo, Salvatore Perdichizzi, Emma C. Manno, Francesca Stoppa, Corrado Cecchetti

**Affiliations:** ^1^Pediatric Emergency Department Pediatric Intensive Care Unit, Bambino Gesù Children's Hospital, Institute for Research and Health Care (IRCCS), Rome, Italy; ^2^Clinical Immunology and Vaccinology Unit, Pediatric Academic Department (DPUO), Bambino Gesù Children's Hospital, IRCSS, Rome, Italy; ^3^Division of Nephrology and Dialysis, Department of Pediatrics, Bambino Gesù Children's Hospital, IRCCS, Rome, Italy

**Keywords:** multisystem inflammatory syndrome in children, CytoSorb, cytokine storm, hemoperfusion, children, pediatric intensive care

## Abstract

**Background:** Multisystem inflammatory syndrome in children (MIS-C) has emerged during the COVID-19 pandemic as a new SARS-CoV-2-related entity, potentially responsible for a life-threatening clinical condition associated with myocardial dysfunction and refractory shock.

**Case:** We describe for the first time in a 14-year-old girl with severe MIS-C the potential benefit of an adjuvant therapy based on CytoSorb hemoperfusion and continuous renal replacement therapy with immunomodulatory drugs.

**Conclusions:** We show in our case that, from the start of extracorporeal blood purification, there was a rapid and progressive restoration in cardiac function and hemodynamic parameters in association with a reduction in the most important inflammatory biomarkers (interleukin 6, interleukin 10, C-reactive protein, ferritin, and D-dimers). Additionally, for the first time, we were able to show with analysis of the sublingual microcirculation a delayed improvement in most of the important microcirculation parameters in this clinical case of MIS-C.

## Background

Multisystem inflammatory syndrome in children (MIS-C) has emerged during the coronavirus disease 2019 (COVID-19) pandemic as a new severe acute respiratory syndrome coronavirus 2 (SARS-CoV-2)-related entity. MIS-C is defined as a clinically severe illness requiring hospitalization with fever, elevated inflammatory biomarkers, and multisystem organ dysfunction in the setting of recently proven or probable SARS-CoV-2 infection and in the absence of an alternative credible explanation (1). Myocardial dysfunction is observed in 80% of the pediatric population with MIS-C, with the majority of cases (68%) requiring intensive care admission: 63% of cases show need for inotropic/vasopressor support, with the main reason for invasive mechanical ventilation (18%) being hemodynamic failure (2). Extracorporeal membrane oxygenation (ECMO) has been documented in 4% of cases, and the observed mortality is 1.5% (2). The pathophysiological mechanism of MIS-C is still unclear (3), but authors have suggested the hypothesis of a delayed cytokine storm driven by the ability of coronavirus to block type I and type III interferon responses (4, 5). Hence, the scientific community has suggested the use of immunomodulatory therapies ranging from corticosteroids and high doses of intravenous immunoglobulin to the interleukin (IL)-1 receptor antagonist on the basis of the clinical phenotype (6).

CytoSorb (CytoSorbents Corporation, Monmouth Junction, NJ, USA) is an extracorporeal adsorber and, in the last 10 years, has been described as an important tool for managing hypercytokinemia in intensive care with the objective of restoring immune homeostasis (7). Recently, papers have confirmed the efficacy and safety of this device in different life-threatening cytokine storm-related clinical conditions (8–10). We herein report, to the best of our knowledge, the first clinical case describing the successful approach of hemoperfusion with CytoSorb in a 14-year-old girl affected by MIS-C SARS-CoV-2, characterized by severe myocardial dysfunction, shock, and multi-organ failure.

Although we are aware of the limitations of a single case report, we highlight the potential role of extracorporeal blood purification as an adjuvant therapeutic strategy in children with severe forms of MIS-C.

## Case Presentation

In December 2020, a 14-year-old girl in previous good health was referred to our Pediatric Intensive Care Unit (PICU) with a suspicion for MIS-C and cardiac dysfunction. She had a 20-day history of asymptomatic SARS-CoV-2 infection (rhinitis without fever); however, 5 days prior to ICU admission, and with a negative SARS-CoV-2 nasopharyngeal swab, she developed a high fever (up to 40°C), maculopapular rash and 3 days later was admitted to hospital for ongoing fever and weakness. The diagnosis of MIS-C was confirmed on the basis of the Centre for Disease Control (CDC) definition (1). On admission in the PICU, she displayed mental confusion, tachypnea, and hypotension associated with oliguria and mild fluid overload. Her laboratory assessment revealed the following: C-reactive protein (CRP), 13.52 mg/dl; ferritin, 2,658 ng/ml; lymphocyte count, 0.39 × 10^3^/μl; albumin, 3.2 g/dl; N-terminal fragment B-type natriuretic peptide (pro-BNP), 2,227 pg/ml; troponin, 31.4 pg/ml; platelets, 70,000 × 10^3^/μl; INR, 1.46 s; and D-dimer, 18.9 μm/ml. Echocardiogram confirmed a myocardial dysfunction characterized by an ejection fraction (EF) of 35% and a chest X–ray with diffuse thickening of the peribroncovascular interstitium and bilateral mild pleural effusion.

Due to the low cardiac output syndrome, tracheal intubation and invasive mechanical ventilation were performed. Along with milrinone 0.5 μg kg^−1^ min^−1^, epinephrine was started and titrated to 0.25 μg kg^−1^ min^−1^. Following the increase in inotropes, the hemodynamic parameters normalized (cardiac index, 2.5–3.0 L min^−1^ m^−2^; systemic vascular resistance index, 2,000–2,400 DS*m^2^/cm^2^); however, we observed a rapid and progressive increase in lactate (increasing rate, >0.5 mmol/l*h; maximum, 10.8 mmol/l). Due to worsening of the metabolic acidosis and the hyperlactatemia, we decided to start continuous renal replacement therapy (CRRT) with a standard hemofilter (ANST69) in continuous venovenous hemodiafiltration (CVVHD) mode using pre-filter reinfusion and an effluent dose of 2,000 ml/h/1.73 m^2^. Due to the hyper-inflammation origin of MIS-C, CytoSorb was also used following the therapeutic recommendations from the Food and Drug Administration for cytokine storm in COVID-19 (11), although our patient was an adolescent weighing 45 kg, but younger than 18 years. Four CytoSorb cartridge columns were used. The first two were changed every 12 h and then the next two were changed every 24 h. The ANST69 filter was changed every 72 h. Pharmacological therapies with methylprednisolone 2 mg kg^−1^ day^−1^ times per day, immunoglobulin 2 g/kg, and low-molecular-weight heparin (LMWH) 100 IU/kg q24h were started. She received anakinra (100 mg q6h) 24 h before PICU admission. Then, this IL-1 receptor antagonist was withheld and then restarted 24 h before the end of blood purification, with the objective of supplementing the hemoperfusion with immunomodulatory pharmacotherapy.

**Figure 1:**
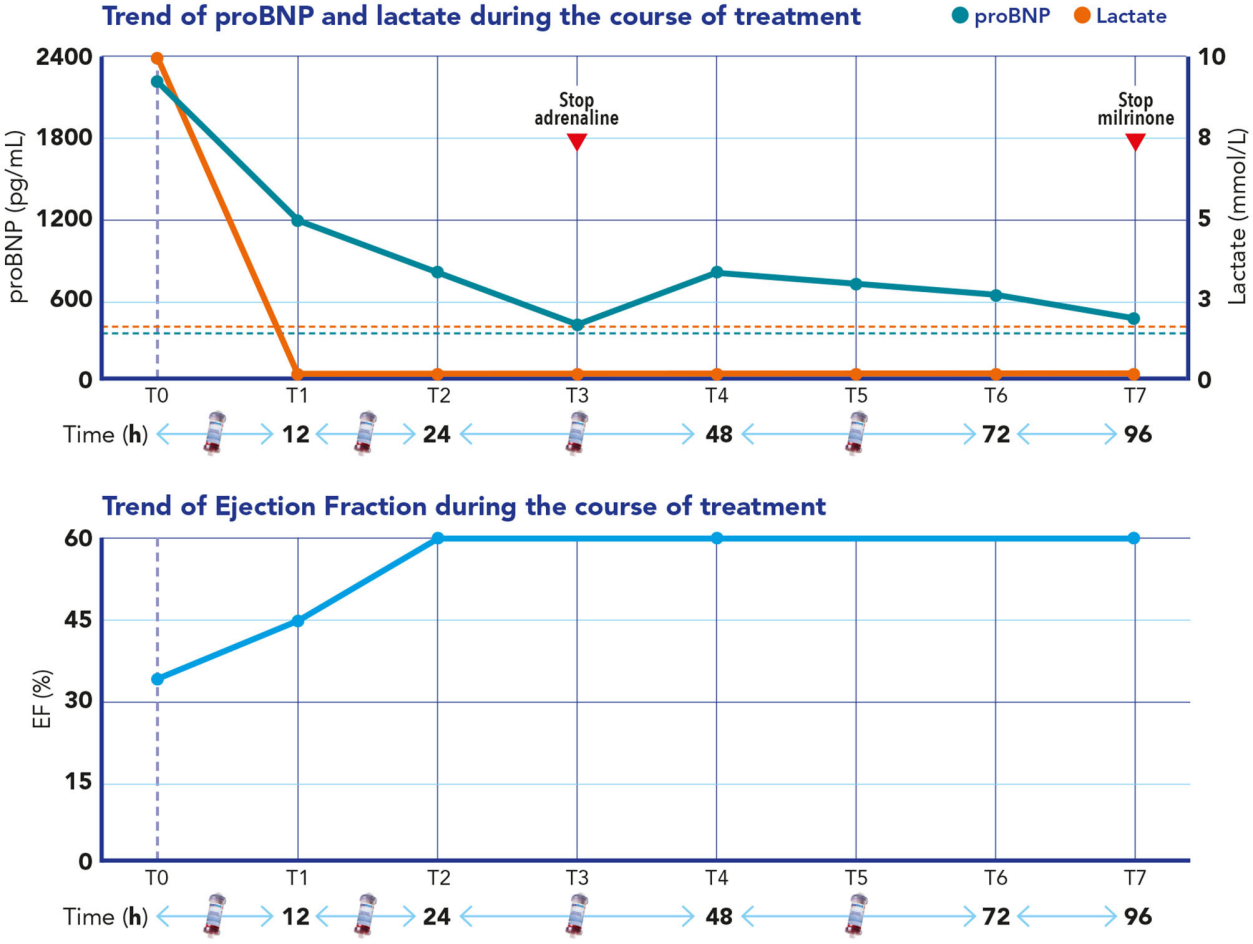
Upper section: Time course of B-type natriuretic peptide (pro-BNP) and lactate during extracorporeal blood purification treatment (EBPT). Blue dashed line refers to the normal value of pro-BNP (<360 pg/ml); *red dashed line* refers to the normal value of lactate (<2 mmol/L). Lower section: Time course of the ejection fraction during EBPT and in the following days after stopping adrenaline, milrinone, and hemoperfusion.

Blood purification was able to control the hyperlactatemia, reaching normal lactate blood levels 3 h after the start of the blood purification. An echocardiogram performed 12 h after the start of the blood purification showed an improvement of her ejection fraction (EF = 45%) and complete normalization by 24 h (EF = 60%) (Figure 1). Pro-BNP progressively improved over the following 3 days (Figure 1), and epinephrine infusion was able to be progressively reduced and totally stopped on day 3 and milrinone on day 7.

**Figure 2:**
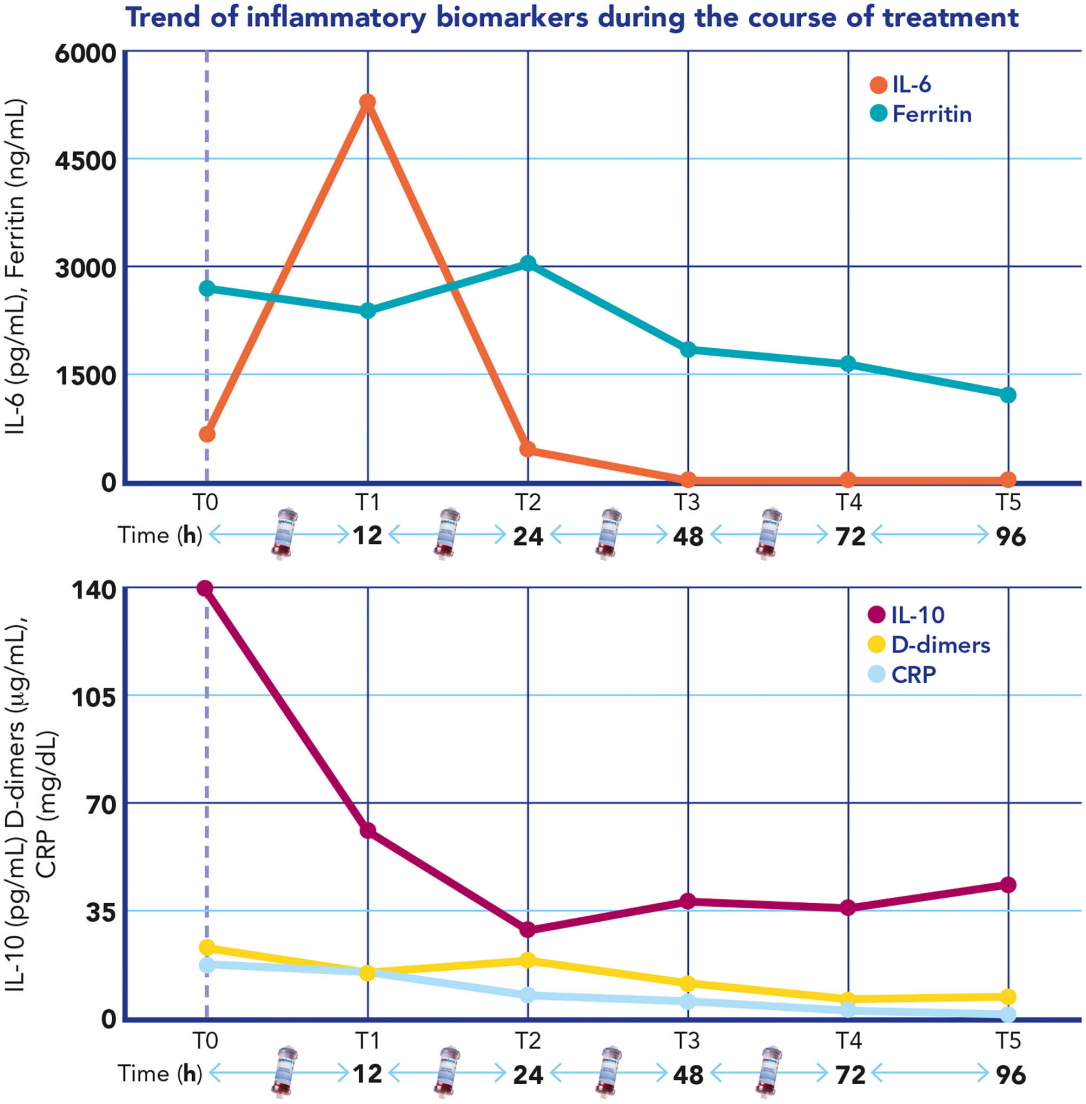
Time course of interleukin 6, interleukin 10, C-reactive protein (CRP), D-dimers, and ferritin during extracorporeal blood purification treatment.

The D-dimer, CRP, and ferritin all decreased as the ejection fraction improved, and vasopressors were weaned off (Figure 2). The same time course was observed for IL-6 and IL-10 blood levels that were measured every 24 h during the blood purification treatment (Figure 2).

**Figure 3:**
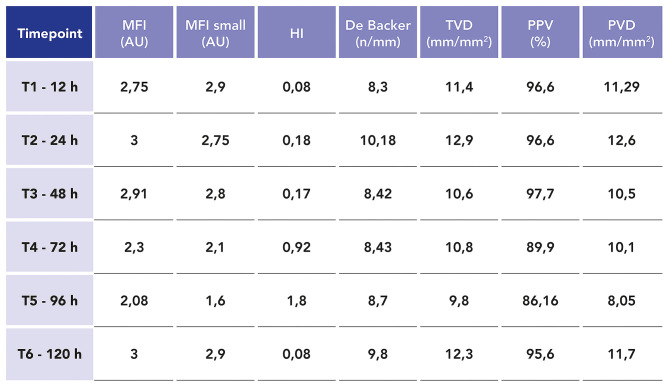
Values of the most important microcirculation parameters related to microvascular flow and capillary density at different time points during extracorporeal blood purification treatment: microvascular flow index (*MFI*), mean flow index small vessels (*MFI small*), heterogeneity index (*HI*), De Backer score, total small vessel density (*TVD*), proportion of perfused vessels (*PPV*), and perfused microvascular density (*PVD*).

We carried out sublingual microcirculation (SM) analysis with the objective of studying the endothelial damage caused by SARS-CoV-2. Five videos were performed for every time point using a handheld vital microscope (HVM) based on incident dark-field microscopy imaging (Braedius Medical, Huizen, The Netherlands). Videos were captured every 24 h from the start of blood purification for the following 6 days. The three best quality videos were chosen for every time point (12, 13). The videos were analyzed offline with dedicated software (Analysis Manager V2; Braedius Medical, Huizen, the Netherlands) (14) and by eye by two independent operators (13, 15). The following parameters were calculated: De Backer score (15), total small vessel density (TVD), proportion of perfused vessels (PPV), and perfused microvascular density (PVD). Semi-quantitative analysis of the microcirculatory flow was performed as previously described by Boerma et al. (16). Each image was divided into four equal quadrants, and for each one, a quantification of flow was scored (no flow: 0; intermittent flow: 1; sluggish flow: 2; continuous flow: 3). Determination of the microvascular flow index (MFI) was based on the predominant type of flow in each quadrant and averaged over the values obtained in each one. We also calculated the heterogeneity index (HI) following the method of Trzeciak et al. (17) based on the MFI and PPV values. Our patient showed, in the first 4 days (96 h), significant SM alterations: the MFI both in all and in small vessels showed, at different time points, a value <2.75 AU, which is correlated with a worse outcome in intensive care (16, 17). The other microcirculation density parameters evidenced in the same way significant alterations at different time points when compared with the normal values observed in this type of analysis (TDV < 16–22 mm/mm^2^, PPV < 95–100%, PVD = 10–18 mm/mm^2^, and De Backer score = 10–12 n/mm) (14). As shown also by an increase of HI (>0.05), a heterogeneity in the microcirculatory perfusion with obstructed capillaries next to capillaries with flowing red blood cells was observed (13, 15–17). In Figure 3, we report the microcirculation parameters at different time points.

**Figure 4:**
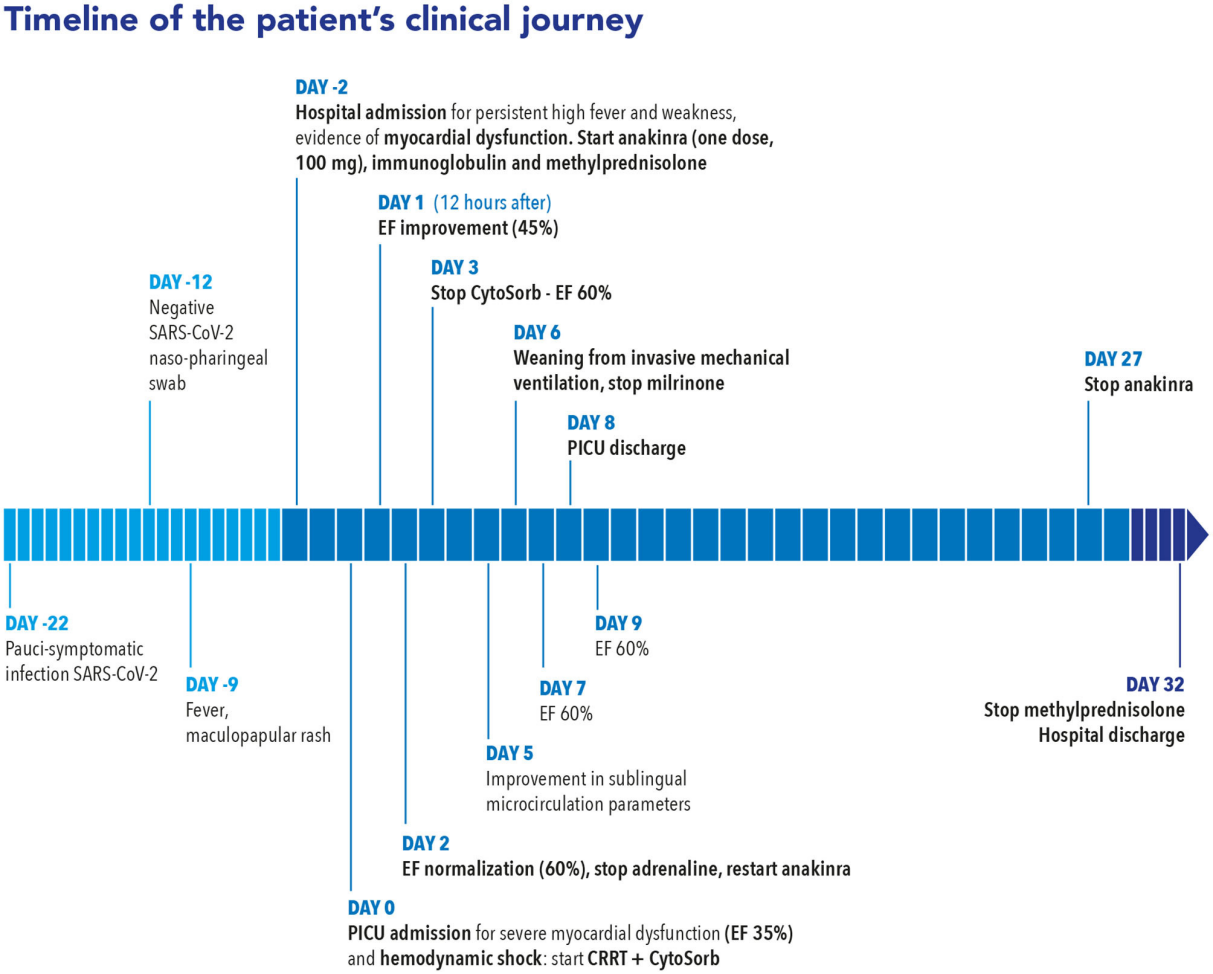
Clinical timeline of a 14-year-old girl diagnosed with SARS-CoV-2-related multisystem inflammatory syndrome with severe myocardial dysfunction and refractory shock. The timeline shows the time course of the clinical evolution and the timing of the multimodal therapeutic approach based on immunomodulatory drugs and extracorporeal blood purification with continuous renal replacement therapy (CRRT) plus CytoSorb hemoperfusion.

SARS-CoV-2 reverse transcription PCR (RT-PCR) was negative on nasopharyngeal, conjunctival swabs and on fecal and urine samples, and was repeated every 48 h confirming the same negative results. A serological test for SARS-CoV-2 immunoglobulin G (IgG) returned a positive result (71 AU; positive reference range, >15 AU/ml). A microbiological workup was negative, including blood, throat, sputum, urine, and stool culture; respiratory and stool viral panel; and blood viral panel including human immunodeficiency virus, hepatitis B virus, hepatitis C virus, cytomegalovirus, Epstein–Barr virus, parvovirus B19, herpes simplex virus 1 and 2, human herpes virus 6, and adenovirus. She continued to improve and, when reassessed on day 15, had a normal cardiac function, so was able to be progressively weaned from the corticosteroids and anakinra therapy. Figure 4 shows a timeline of our patient's clinical journey.

## Discussion

The goals of treatment for MIS-C are to shut off the overshooting systemic inflammation and restore organ function in order to decrease mortality and to reduce long-term sequelae. A stepwise approach to immunomodulatory treatment is recommended by the American College of Rheumatology (ACR) (18). Intravenous immunoglobulin (IVIG) and/or glucocorticoids alone, or in combination with anakinra, are the most commonly used immunomodulators and should generally be tried first. Treatment should be dictated by phenotype (6), but also by the severity of the clinical condition. In contrast to acute COVID-19 infection in children, MIS-C appears to be a critical condition, with 68% of cases requiring critical care support (2).

We have, to the best of our knowledge, described for the first time the potential role of hemoperfusion with CytoSorb in children with severe MIS-C. The efficacy of CytoSorb has already been described in other clinical settings characterized by life-threatening cytokine storm (8–10); recently, the FDA has suggested the use of CytoSorb in clinical pictures of cytokine storm related to COVID-19 (11).

In our case, we observed a rapid recovery in the myocardial dysfunction associated with a progressive reduction in pro-BNP, lactate, and other inflammatory biomarkers. This clinical experience shows that maybe “intensive” hemoperfusion could be an advantageous adjuvant therapy in patients with refractory shock and multiple organ dysfunction in MIS-C, potentially avoiding the need for ECMO and without interfering with the most common immunomodulatory therapies. The time course of cytokines at different time points shows significant cytokine removal by 24 h from the start of the blood purification, even in the absence of anakinra in the first 48 h of blood purification treatment. Furthermore, the cytokine blood levels at time points 4 and 5 exclude any cytokine rebound after stopping the hemoperfusion, confirming definitive control of the cytokine storm. On the other hand, we would highlight that hemoperfusion has been combined with several other immunomodulatory treatments (steroids, IVIG, and anakinra), which certainly had a significant impact on the hyper-inflammatory state and on the clinical course of our patient. Furthermore, in our clinical case, the extracorporeal blood purification approach was based on hemoperfusion plus CRRT. This latter extracorporeal blood purification technique has provided, in our opinion, some important benefits in addition to hemoperfusion: firstly, CRRT helped with the efficacious and prompt management of hyperlactatemia and of acid–base imbalance; in addition, CRRT also helped control the fluid overload shown by the patient on PICU admission.

For the first time, we have monitored not only the mediators of inflammation but also endothelial damage, observed through SM analysis, in MIS-C. Microcirculation of the sublingual mucosa has been shown to be a good representative of other microvascular beds under a variety of pathophysiological conditions (19–21), and several studies have demonstrated that persistent microcirculatory disturbances could be a sign of worse outcomes in critically ill pediatric patients (22).

We are able to show that the microcirculation is impaired in MIS-C, highlighting a lack of “hemodynamic coherence” characterized by a restoration of the macro-circulatory hemodynamics without parallel improvement in the microcirculation (23) in the first 96 h of the clinical course. In fact, despite hemodynamic improvement and rapid weaning of inotropes on the third day, the principal microcirculatory parameters only improved after 96 h (fifth day), showing, in this clinical case, the slower restoration of the endothelial damage. Similar results have been found in previous studies (24, 25) on pediatric patients with septic shock, where a lack of hemodynamic coherence has been associated with an increase in mortality in cohorts of patients analyzed by SM analysis.

## Conclusion

MIS-C is a severe complication of COVID-19 in children and adolescents. Although the mortality of MIS-C is low, this syndrome is associated with severe myocardial dysfunction and multi-organ failure. In our report, we show the potential benefits of hemoperfusion with CytoSorb in severe MIS-C. The aim of our paper has been not to establish a relationship between the use of CytoSorb and the clinical improvement of our patient, but to share with the scientific community the potential benefit of this adjuvant therapy in a severe case of MIS-C in combination with other immunomodulatory therapies. We show a lack of hemodynamic coherence between macro- and microcirculation in MIS-C, with late restoration of the microcirculation despite the more rapid improvement in the hemodynamic parameters associated with the blood purification treatment. Other authors have described in MIS-C not only hypercytokinemia but also endothelial dysfunction (26), and we provide further evidence to this hypothesis.

Patients with severe multisystem involvement, particularly those with shock, should receive prompt immunomodulatory treatment. In this scenario, CytoSorb could also represent a valid adjuvant therapeutic option.

## Data Availability Statement

The raw data supporting the conclusions of this article will be made available by the authors, without undue reservation.

## Ethics Statement

The studies involving human participants were reviewed and approved by IRCCS Bambino Gesù Children's Hospital. Written informed consent to participate in this study was provided by the participant's legal guardian/next of kin. Written informed consent was obtained from the minor(s)' legal guardian/next of kin for the publication of any potentially identifiable images or data included in this article.

## Author Contributions

GB drafted the manuscript. All authors were involved in the clinical management, analyzed and interpreted the data, and discussed the results, read and approved the final manuscript.

## Conflict of Interest

The authors declare that the research was conducted in the absence of any commercial or financial relationships that could be construed as a potential conflict of interest.
